# *Burkholderia pseudomallei*-derived miR-3473 enhances NF-κB via targeting TRAF3 and is associated with different inflammatory responses compared to *Burkholderia thailandensis* in murine macrophages

**DOI:** 10.1186/s12866-016-0901-6

**Published:** 2016-11-28

**Authors:** Yao Fang, Hai Chen, Yi Hu, Qian Li, Zhiqiang Hu, Tengfei Ma, Xuhu Mao

**Affiliations:** 1 Department of Clinical Microbiology and Immunology of Southwest Hospital and the College of Medical Laboratory Science, Third Military Medical University, No. 30 Gaotanyan Street, Shapingba District, Chongqing, 400038 People’s Republic of China; 2 PLA 161 Hospital, Wuhan, 430014 People’s Republic of China; 3 Department of Clinical Laboratory, People’s Hospital of Sanya, Sanya City, Hainan Province 572000 People’s Republic of China

**Keywords:** *Burkholderia pseudomallei* (Bp), *Burkholderia thailandensis* (Bt), TNF-α, Apoptosis, miR-3473, TRAF3, NF-κB

## Abstract

**Background:**

*Burkholderia pseudomallei* (Bp) is the causative agent of melioidosis, a kind of tropical disease. *Burkholderia thailandensis* (Bt), with a high sequence similarity to Bp, is thought to be an avirulent organism. Since there are numerous similarities between Bp and Bt, their differences in pathogenesis of host response and related mechanism are still undermined. In recent years, microRNAs have been researched in many diseases, but seldom involved in bacterial infection, bacteria-host interaction or explaining the differences between virulent and avirulent species.

**Results:**

We found that Bp and Bt had similar phenotypes in terms of intracellular replication, dissemination (reflected by multinucleated giant cell formation), TNF-α release and apoptosis in RAW264.7 macrophages or TC-1 pulmonary cell but in different level. Especially, at the late infection phases (after 12 h post infection), Bp showed faster intracellular growth, stronger cytotoxicity, and higher TNF-α release. After microRNA array analysis, we found some microRNAs were significantly expressed in macrophages treated by Bp. miR-3473 was one of them specifically induced, but not significantly changed in Bt-treated macrophages. In addition, TargetScan suggested that miR-3473 possibly target *TRAF3* (TNF receptor-associated factor 3), a well-known negative regulator of the NF-κB pathway, which was probably involved in the TNF-α induction and apoptosis in cells with Bp infection. In vivo, it was found that miR-3473 expression of total lungs cells from Bp-treated was higher than that from Bt-treated mice. And miR-3473 inhibitor was able to decrease the TNF-α release of mice and prolong the survival of mice with Bp infection.

**Conclusion:**

In sum, miR-3473 plays an important role in the differential pathogenicity of Bp and Bt via miR-3473-*TRAF3*-TNF-α network, and regulates TNF-α release, cell apoptosis and animal survival after Bp treatment. In this study, we have found a specific microRNA is related to bacterial virulence and provide insight into the mechanism for host-bacteria interaction, which suggests that potential oligonucleotides should be applied against bacterial infection.

*Burkholderia pseudomallei* is an opportunistic pathogen and the causative agent of melioidosis, which is well-known in tropical zones and has spread to boarder areas. In contrast, *Burkholderia thailandensis* is less known and even considered an avirulent organism before, but now it has been proved to be associated with human pulmonary cystic fibrosis, and also infectious to mice in certain circumstance. Though there are some similarities between these two species, the mechanism behind these difference is still underground and may be the key to understand their pathogenesis, vaccine development and drug design. Apart from some secreted virulence factors and bacterial structure proteins, microRNAs have been classified as important factors in the pathogenesis of some bacterium, such as *Burkholderia pseudomallei* and *Salmonella typhi*, which have been proved to manipulate host autophagy or innate immunity through microRNA-mRNA network. In this study, a specific microRNA, miR-3473, has been identified to associate with the pathogenesis of *Burkholderia pseudomallei* in macrophages through a microRNA-*TRAF3*-TNF-α network, and influence the survival of mice with Bp infection through regulating TNF-α release. Though there may be other influence factors involved in differences between Bp and Bt, miR-3473 has provided a novel explanation and shed a light on the mechanism for bacterial infection from a view of microRNA.

## Background

*Burkholderia pseudomallei* (Bp) is the causative agent of the human disease melioidosis, which is endemic in southeast Asia and northern Australia, with manifestations ranging from fever to pneumonia to life-threatening sepsis. Bp causes hundreds of human infections and a heavy economic burden in these regions [1]. *Burkholderia thailandensis* (Bt) is first identified as a Bp-like species since their genomes are highly syntenic and their intracellular behaviours are similar [2–4]. *Burkholderia thailandensis* is basically considered to be an avirulent organism, a possible substitute for Bp and even a candidate for an attenuated vaccine [5, 6].

There are some similarities between Bp and Bt in terms of the ability of intracellular replication, cytokines induction, and immune cell differentiation [7, 8]. Bt has exhibited reduced replication in human macrophages and deficient invasion into epithelial cells compared to Bp [9, 10]. In vivo, it requires 2 × 10^2^-fold more Bt by intranasal inoculation to infect and induce inflammation in C57BL/6 mice than Bp, and an inhalation model of Bt has been successfully established with an infection dosage of 3 × 10^4^ cfu/lung [6]. Bt infections have been also associated with human pulmonary cystic fibrosis [11, 12]. To date, it needs more research into Bt or Bp-specific pathogenesis or mechanism for their different phenotypes.

Macrophage modeling has been applied in studies with *Burkholderia* spp, as well as other pathogens (e.g. *Yersinia pestis*, *Yersinia pseudotuberculosis*, *Francisella tularensis*, and *Bacillus anthracis*). It provides a protected niche for these intracellular pathogens against host immune responses, including Bp or Bt strains. Increasing investigations have beenfocused on the mechanisms of *Burkholderia* pathogenesis within macrophages and exploring the application of specific macrophage phenotype as a potential biomarker for *Burkholderia* related infections. A previous microarray analysis has indicated that *bcl2* gene expression was 2-fold higher in the Bp-infected A549 cells than that in the Bt-infected A549 cells [13]. Clinical studies have suggested that elevated TNF-α, IL-10, IL-1β, IL-8, IL-6 and IFN-γ have been associated with mortality among patients with melioidosis [14]. Specifically, TNF-α has been even considered as a ‘death factor’ in melioidosis, closely related with the outcome of clinical melioidosis [15].

microRNAs are small non-coding RNA molecules found in plants, animals, and even microorganisms and have been involved in many diseases, including inherited disease, cancer, metabolic disease, heart disease, kidney disease, nervous disease, obesity and viral infection [16, 17]. Recently, microRNAs have also been reported to associate with the biochemical process of certain pathogens, such as *Burkholderia pseudomallei* and *Salmonella typhi-*related autophagy or innate immunity [18, 19]. Additionally, dual RNA sequencing has revealed that some noncoding RNAs in *Salmonella* are correlated with the expression of virulence genes related with bacterial infection and host response [20].

In our previous microRNA-mRNA array analysis, we have found that Bp infection elevates the levels of apoptotic inflammatory cytokines, in particular TNF-α, with the fold-change value of 74.94 at 24 hpi (hours post infection, compared to negative control), which was much more higher than that of Bt. It was similar to the apoptosis induced by Bp and Bt. Since several microRNAs significantly changed as the same time, we wondered whether there are some microRNAs specifically induced by Bp infection or related to host responses, like TNF-α release or apoptosis. We hypothesized that microRNA-mRNA regulation network could play an important role in the Bp and Bt-induced cell or animal phenotypes and might provide a novel mode of research on virulent and avirulent bacterial strain.

## Methods

### Bacterial strains and cells

The clinical strain *Burkholderia pseudomallei* BPC006 was obtained from the Hainan province, China, and was completely sequenced [21]. *Burkholderia thailandensis* E264 (ATCC® 700388) was purchased from American Type Culture Collection (ATCC, Maryland, USA). Bacteria were grown overnight on LB agar or in LB broth shaken at 200 rpm at 37 °C for 16 h prior to use.

RAW264.7 cell line (ATCC® TIB-71) was purchased from ATCC, and the TC-1 cell line was a gift from Professor Guansong Wang (Institute of Respiratory Diseases, Xinqiao Hospital, Third Military Medical University). The cells were maintained in DMEM or RPMI-1640 supplemented with 10% heat-inactivated foetal bovine serum (FBS) (Gibco-BRL, New York, USA) at 37 °C in a humidified atmosphere of 95% oxygen and 5% carbon dioxide.

### Infection, intracellular multiplication, MNGC formation and apoptosis assay

RAW264.7 cells were seeded at a concentration of 5 × 10^5^ cells per well into 12-well plates 12 hpi. Cells were infected with bacteria grown for 16 h (stationary phase) at the indicated multiplicity of infection (MOI) ratios and incubated at 37 °C in 5% CO_2_ for 2 h. The culture medium was then removed and replaced with medium containing 250 μg/mL kanamycin (TIANGEN, Beijing, China) to prevent any further extracellular bacterial replication. Incubation was continued up to 28 hpi, depending on experimental design. After the infection assays, macrophages were washed and permeabilized using 1% Triton X-100 at different intervals. Intracellular bacteria that were liberated were quantitated by dilution and plating on trypticase soy agar. The number of bacterial colonies was counted after 24 to 36 h of incubation.

MNGC (Multinucleated giant cell) formation was shown by Giemsa staining. Cells were seeded and grown overnight on glass cover slips. At different time points after infection with Bp, the cover slips were washed with PBS, fixed with 1% paraformaldehyde for 10 min, and then washed with PBS for 5 min. The cover slips were air dried before staining with the Giemsa stain. For evaluation of MNGC formation, at least 1,000 nuclei per cover slip were counted, and the percent of MNGC formation was calculated as follows: (number of nuclei within multinucleated cells/total number of nuclei counted) × 100%.

The cells were trypsinized with 0.5 ml of 0.25% trypsin for 3 min, collected, and resuspended in 1 ml of PBS. The cells were then treated with Annexin V-FITC/PI kit according to the manufacturer’s instructions and detected using flow cytometry (BD FACScan flow cytometer). Apoptotic cells were classified as normal cells, early apoptosis cells, late apoptosis cells and necrosis cells.

### Plasmids construction, transfection and luciferase assay

The plasmids pMIR-Report and pRL-TK were purchased from Ambion. The fragments of the TRAF3 3ˈ-UTR containing the miR-3473 target site were amplified from genomic DNA (primers details in Table 1) and cloned into the pMIR-Report plasmid downstream of a reporter synthetic *Renilla* luciferase gene (hRluc) using SpeI and HindIII. Firefly luciferase located downstream of the 3ˈ-UTR fragment served as a transfection internal control. To generate plasmids with one or more mutations in the binding site for miR-3473, the seed regions were mutated from *TCTCTCCA* to *TGTTTGCA* at 1186–1193 of the 3ˈ-UTR and synthesized by Sangon (Shanghai, China) followed by cloning into the pMIR-Report plasmid.

**Table 1:** Primers used in this study

Target gene	Sequence	Length (nt)	Note
*TNF*-α*-F*	*CCCCTCAGCAAACCACCAAG*	20	
*TNF*-α*-R*	*CTTGGCAGATTGACCTCAGC*	20	
*TRAF3-F*	*GAGCAAGGAGGCTACAAGGAG*	21	[32]
*TRAF3-R*	*CATGCAGCTCTCGCAGAAC*	19	
*Actin-F*	*TGGCACCCAGCACAATGAA*	19	[32]
*Actin-R*	*CTAAGTCATAGTCCGCCTAGAAGCA*	25	
*P-*_*TRAF3-overexpress*_ *-F*	*ATGGAGTCAAGCAAAAAGATGGA*	23	
*P-*_*TRAF3-overexpress*_ *-R*	*GGGGTCAGGCAGATCCGA*	18	
*miR-3473-TRAF3-F*	*CTAGTGGTTCTAGAAAGTGTCAGTTTAACCAGA* *TCTCTCTCCACCACCAGAACTTTGTCTCTGCCA*	60	Sangon
*miR-3473-TRAF3-R*	*AGCTTGGCAGAGACAAAGTTCTGGTGGTGGAG* *AGAGATCTGGTTAAACTGACACTTTCTAGAACCA*		
*miR-3473-TRAF3-mut-F*	*CTAGTGGTTCTAGAAAGTGTCAGTTTAACCAGA* *TCTGTTTGCACCACCAGAACTTTGTCTCTGCCA*	60	
*miR-3473-TRAF3-mut -R*	*AGCTTGGCAGAGACAAAGTTCTGGTGGTGCAA* *ACAGATCTGGTTAAACTGACACTTTCTAGAACCA*		

RAW264.7 cells were seeded in 24-well plates (1 × 10^5^/well) one day before transfection. Cells were transfected with the indicated vectors for each experiment using Lipofectamine 2000 (Life Technologies, USA). Luciferase activity was determined using the dual luciferase assay system (Promega, USA) according to the manufacturer’s instructions.

### Quantitative RT-PCR (qRT-PCR)

To confirm the validity of the microarray, the expression levels of TNF-α, TRAF3 and miR-3473 were detected by qRT-PCR. Given the replacement of medium during infection, qRT-PCR was used to detect TNF-α mRNA expression rather than to measure the concentration of TNF-α in supernatant by ELISA. Total RNA was extracted from cells using the phenol-chloroform method and was subsequently reverse transcribed using the PrimeScript RT reagent kit (TaKaRa, Japan). The cDNA preparations were stored at −20 °C until PCR amplification. The qPCR for *TNF-α* and *TRAF3* was performed using the primers in Table 1. Reactions containing SYBR Green I MasterMix (TOYOBO, Japan) were prepared in final volumes of 20 μL in 96-well plates. The amplification protocol consisted of an initial hot start (95 °C for 1 min), followed by 40 cycles of 95 °C for 10 s, 55 °C for 5 s and 72 °C for 20 s, and ended with a melt analysis by ramping amplicons from 60 °C to 95 °C with 0.5 °C increments. The Bio-Rad IQ5 (Bio-Rad Laboratories, Inc.) System was used for the PCR step and data analysis. We evaluated the mRNA level of TNF-α rather than its protein level because the cell medium was replaced with antibiotic-containing medium after 1 to 2 h of incubation with bacteria to avoid interfering with the concentration of TNF-α in supernatant.

miR-3473 was measured using TaqMan microRNA assays (Ambion, 4465407) in a Bio-Rad IQ5 detection system. The reactions were performed using the following parameters: 95 °C for 5 min followed by 40 cycles of 95 °C for 20 s and 60 °C for 30 s. U6 small nuclear RNA (001973) was used as an endogenous control for data normalization. Relative expression was calculated using the comparative threshold cycle method.

### Protein extraction and western blot analysis

Twelve hours prior to infection, RAW264.7 cells were seeded in a 35-mm dish (2 × 10^6^ cells per dish) and infected for 1 h with the Bp strain (BPC006) or Bt E264 at an MOI of 10. Proteins of RAW264.7 cells were prepared by Protein Extraction Reagent (Thermo Fisher, USA) according to manufacturer’s instructions. Protein content was determined using a NanoDrop-1000 (Thermo Fisher, USA). Equal amounts of protein were separated by SDS-PAGE and transferred onto PVDF membranes (Millipore, Massachusetts, USA) by electroblotting. Membranes were blocked with 5% skim milk in PBST for one hour at room temperature and subsequently incubated overnight at 4 °C with a rabbit anti-TRAF3, NF-κB p65 (D14E12), phospho-NF-κB p65 (Ser536) and Actin (Cell Signalling Technology, Boston, USA) in PBS (pH 7.6), 5% (w/v) BSA and 0.1% (v/v) Tween-20. Horseradish peroxidase (HRP)-conjugated anti-rabbit IgG (Jackson Immuno-Research Lab, Pennsylvania, USA) was used as a secondary antibody for one hour at room temperature. The Gel Image system (Bio-Rad, USA) and Image J programme were used for detection.

### Animal experiments

Specific-pathogen-free BALB/c mice were obtained from Ding guo Chang sheng Biotechnology (Beijing, China). Animals were housed in individual ventilation cages and air was filtered by high efficiency filters. Euthanasia was accomplished with CO_2_. Experiments associated with bacteria or infected-mice were conducted in Biological safety protection third-level laboratory (BSL-3) in Beijing Institute of Microbiology and Epidemiology. Mice were inoculated with drops containing determined number of bacteria in nasal cavity, approximately 25–50 μL every mice. Animals were examined daily for illness or death.

Total lung cells were extracted from fresh lung tissue of Bp or Bt-infected BALB/c mice. Lung tissues were separated quickly and washed with PBS for 5 times. Then the tissue was homogenized in TriZol with a homogenizer. Total RNA was extracted from cells using phenol-chloroform method and prepared for qRT-PCR test. Analysis of miR-3473 was conducted as described above. The levels of serum TNF-α from mice were measured by Elisa Kit (Ding guo Chang sheng, Beijing, China) and conducted as the manufacturer’s introduction. siRNAs or oligonucleotides (miR-3473 mimics, inhibitors, mimic control and inhibitor control) were purchased from Ribobio Corporation (Guangzhou, China) and suspected oligonucleotide was delivered through aerosol inhalation (15 μg in 50 μL PBS/every dose) [22].

### Statistics

Bacterial counts were log-transformed to improve the normality of the data and expressed as the mean ± SEM. Significant differences between two groups were assessed using unpaired t-tests. All data analyses were carried out using Prism version 5.0 (GraphPad Software).

## Results

### Intracellular survival and cytotoxicity of Bp and Bt in murine macrophages

**Fig. 1:**
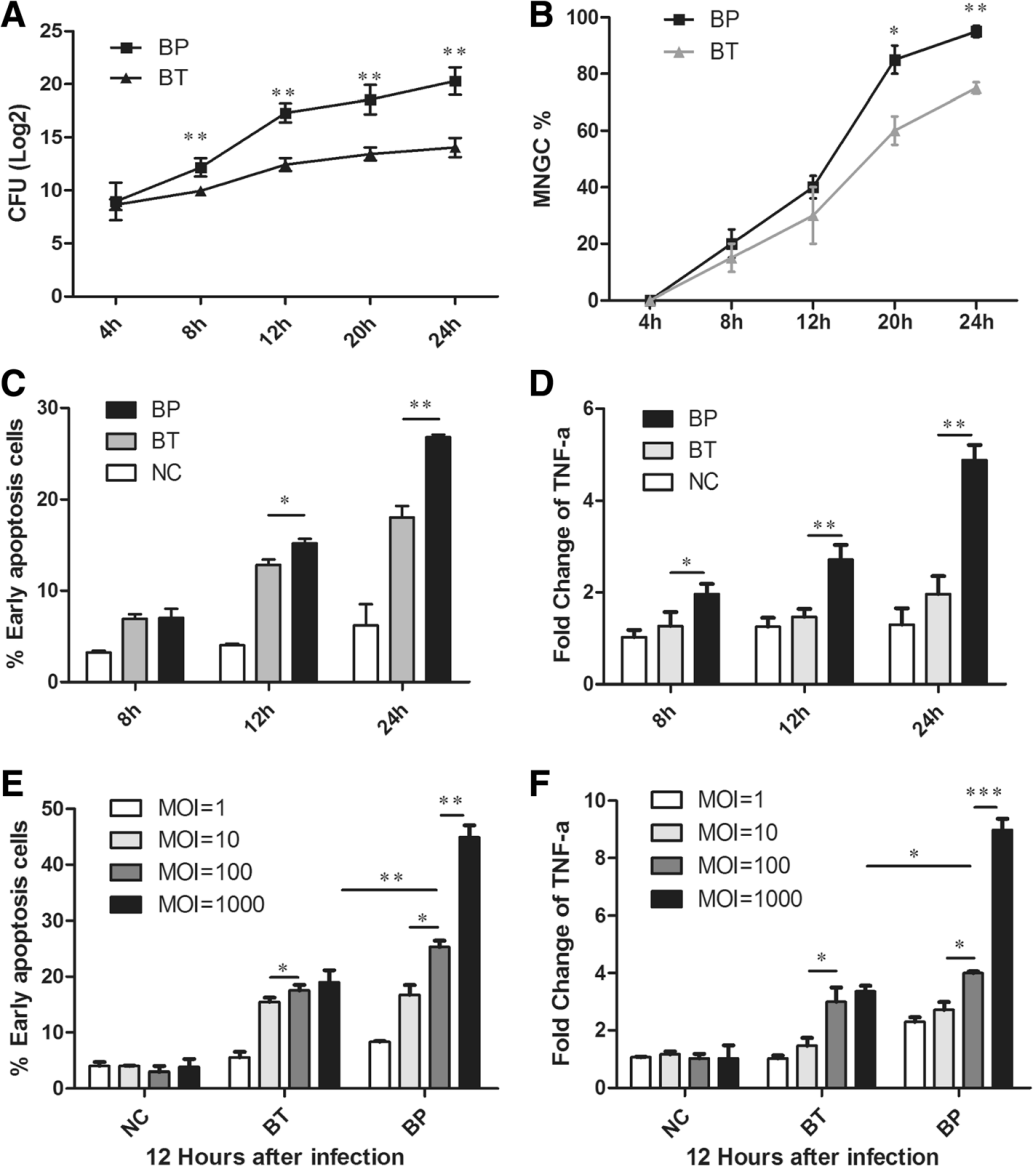
Intracellular survival and cytotoxicity of Bp and Bt in RAW264.7 cell. **a** Intracellular bacteria loads of Bp and Bt were measured at different time (4 h, 8 h, 12 h, 20 h, 24 h, MOI = 10) by gently rupture with 1% Triton X-100. **b** MNGC formation was observed after Bp or Bt infection to RAW264.7 cell at different time (4 h, 8 h, 12 h, 20 h, 24 h, MOI = 10), and MNGC% was calculated (MNGC% = (the number of nuclear of MNGC)/(the number of nuclear of all cells) × 100%). **c** Flow cytometry (FCM) was applied to evaluate cell apoptosis at different time post infection (8 h, 12 h, 24 h, MOI = 10) and early apoptosis cells were counted by gating. **d** mRNA level of TNF-α in RAW264.7 cell was measured by qRT-PCR at different time post infection with *actin* as a reference gene (8 h, 12 h, 24 h, MOI = 10). **e** Early apoptosis cells were counted at 12 h post infection with different dose of Bp or Bt (MOI = 1, 10, 100, 10000). **f** TNF-α mRNA levels were measured at 12 h post infection with different dose of Bp or Bt (MOI = 1, 10, 100, 10000). *P value < 0.05, **P value < 0.01

Bp and Bt showed a similar intracellular growth rate in the early phase (<4 hpi), while Bp grew more rapidly from 16 hpi to 28 hpi, reflecting an accelerating growth rate of Bp in the late infection phase (Fig. 1a). We did not evaluate the intracellular bacterial load after 28 hpi since infected cells began to collapse at that time. Multinucleated giant cells (MNGCs) generally indicate the replication and dissemination of *Burkholderia* species. We found there was no significant difference between that of Bp- and Bt-infected macrophages until 20 hpi (Fig. 1b) and Bt caused an equally high percent (>95%) of MNGC formation after 28 hpi. On the contrary, Bp was showing a relative mild cytotoxicity and growing slowly at the early infection phase (prior to 12 hpi), but followed by an accelerating growth burst after 12 hpi. Apoptosis detection and cytokine expression, particularly for TNF-α, were also the same case. At an early infectious phase (<12 hpi), Bt caused more severe apoptosis than Bp, but this case reversed after 12 hpi (Fig. 1c). TNF-α is considered to be a deleterious cytokine and defined as an important inflammatory marker of apoptosis [15]. We observed that TNF-α expression was always higher in Bp-treated macrophages than that of Bt at various MOI value or infectious phase (Fig. 1d). In addition, MNGC formation and TNF-α expression increased dependent on the dose of Bp infection (Fig. 1e, f).

### miR-3473 was specifically induced in Bp-infected murine macrophages

**Fig. 2:**
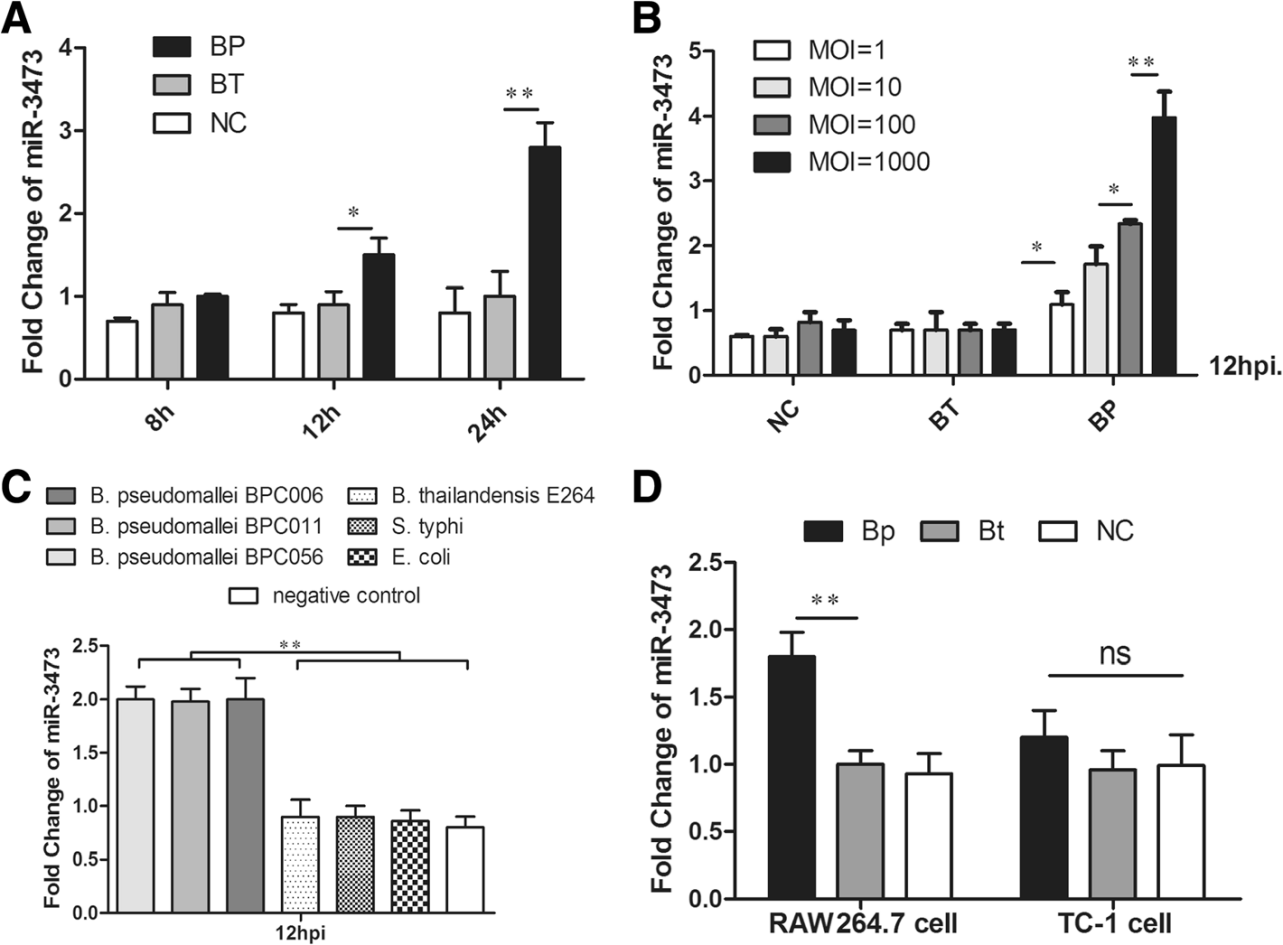
miR-3473 expression at different times post infection in murine macrophages or epithelial cells. **a** qRT**-**PCR analysis of miR-3473 expression in Bp, Bt-infected or uninfected macrophages at different time post infection (8 h, 12 h, 24 h, MOI = 10). **b** miR-3473 expression in Bp, Bt-infected or uninfected macrophages at 12 h post infection with different dose of Bp or Bt (MOI = 1, 10, 100, 10000). **c** miR-3473 expression of macrophages treated with different pathogens (MOI = 10) for 12 h post infection, including *Salmonella typhi, Escherichia coli DH5a,* Bt or 3 strains of clinical Bp (BPC006, BPC011, BPC056, all sequenced by MLST and deposited in http://bpseudomallei.mlst.net/). **d** miR-3473 expression in murine macrophages (RAW264.7 cell line) or epithelial cells (TC-1 cell line) at 12 h post Bp, Bt or no infection (MOI = 10 or 0). *P value < 0.05, **P value < 0.01

Based on microRNA array analysis, several microRNAs were found significantly expressed (fold change value > 1.5) in Bp-infected murine macrophages compared to untreated macrophages, including four up-regulated microRNAs (miR-714: +4.56, miR-3734: +2.31, miR-5105: +1.77, miR-326: +1.60, at 24 hpi) and three down-regulated microRNAs (miR-3082-5p: −2.26, miR-466i-5p: −1.74, miR-574-5p: −1.73, at 24 hpi). But, miR-3473 specifically increased in Bp-infected murine macrophages, rather than that in Bt-treated macrophages and increased significantly at 12 hpi (Fig. 2a). In addition, miR-3473 increased in a dose-dependent manner after Bp infection, but no alteration of miR-3473 in Bt-infected cells was observed, even at a high MOI (Fig. 2b). Moreover, other Gram-negative bacterium, like *Salmonella typhi* and *Escherichia coli DH5α*, were not able to induce miR-3473 significantly (Fig. 2c). We also tested the expression of miR-3473 in TC-1 cell (a type of murine respiratory epithelial cells) treated by Bp, but it did not changed significantly as that in macrophages (Fig. 2d). This indicated that miR-3473 was induced specifically in murine macrophages rather than murine pulmonary cell after Bp infection.

### *TRAF3*, not *TNF-α*, was a direct target of miR-3473

**Fig. 3:**
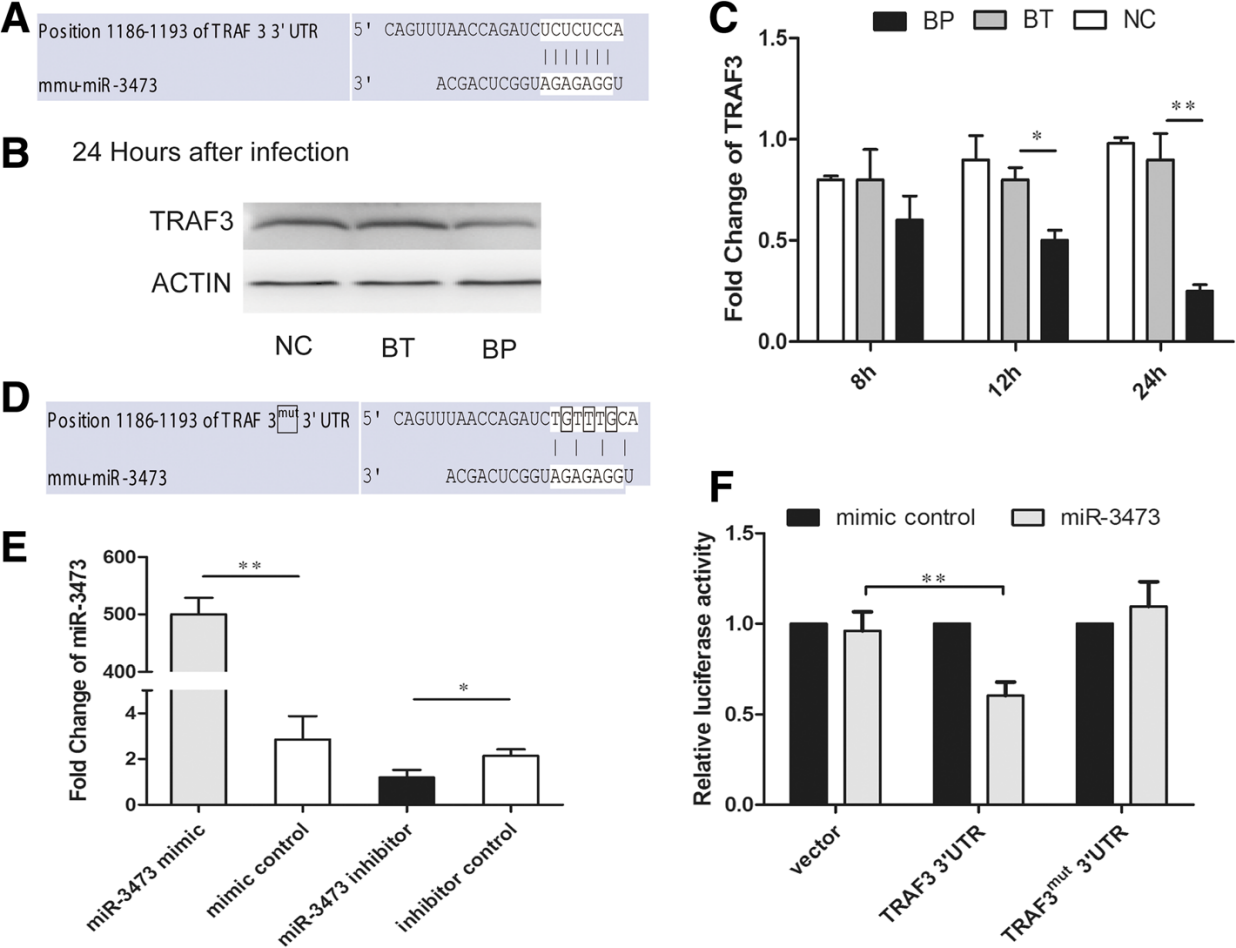
miR-3473 targets TRAF3. **a** The putative binding sites of miR-3473 in the *TRAF3* 3′-UTR are shown (TargetScanMouse 6.2, http://www.targetscreening.org/). **b** TRAF3 protein levels were measured by western blotting in Bp, Bt or uninfected macrophages at 24 h post infection. **c** *TRAF3* mRNA levels were measured in Bp, Bt or uninfected macrophages by qRT-PCR at different time post infection (8 h, 12 h, 24 h) with *actin* as a reference gene. **d** The putative binding sites of miR-3473 in the *TRAF3* 3′-UTR were mutated as shown. **e** qRT-PCR analysis of miR-3473 expression in macrophages transfected with mimic, inhibitor, mimic control or inhibitor control oligonucleotides of miR-3473. **f** Macrophages treated with oligonucleotides (as Figure **e**) were co-transfected with a pMIR-Report-*TRAF3*-3′-UTR-luciferase or pMIR-Report-*TRAF3*-mut-3′-UTR-luciferase or pRL-TK vector as an internal control. After 24 h treatment, cell extractions were analyzed by luciferase reporter assay and values normalized to renilla activity. Data is represented as mean ± SEM from three independent experiments. *P value < 0.05, **P value < 0.01

At first, predictions with TargetScan Mouse 6.2 and miRDB suggested that miR-3473 may be not a regulator of *TNF-α*. While, among the list of targets of miR-3473, TNF receptor-associated factor 3 (*TRAF3*) was included and suggested it should be a potential target of miR-3473 (Fig. 3a). Previous studies have proved that TRAF3 is a key negative regulator of the NF-κB pathway [23, 24]. This way, we hypothesized that miR-3473 regulated TNF-α expression through targeting *TRAF3* rather than direct anchoring.

First, TRAF3 expression in Bp or Bt-treated murine macrophages was analysed by western blotting. TRAF3 reduced significantly and specifically in Bp-infected macrophages (approximately 45% amount of control, 24 hpi) compared to that of Bt-infected or untreated cells (Fig. 3b). *TRAF3* mRNA was detected by qRT-PCR and also showed a significant decrease in Bp-infected macrophages at 12 hpi than that of Bt-treated or untreated cells (Fig. 3c).

To verify that *TRAF3* is a molecular target of miR-3473, a fragment of the 3ˈ-UTR of *TRAF3*, containing the putative miR-3473 binding site (Fig. 3a) or a mutant miR-3473 binding site (Fig. 3c), was individually cloned into a pMIR-Report luciferase plasmid, with pRL-TK as an internal control. A mimic and inhibitor of miR-3473 were transfected into macrophages using Lipofectamine 2000™, and transfection efficiency was confirmed by qRT-PCR (Fig. 3e). After luciferase assay on macrophages which were carrying the 3ˈ-UTR *TRAF3* luciferase reporter, it was found that induction of miR-3473 would significantly inhibit the luciferase activities but there was no inhibition in those macrophages transfected with the mutant 3ˈ-UTR *TRAF3* luciferase reporter (Fig. 3f). It suggested that *TRAF3* (3ˈ-UTR) should be a probable target of miR-3473.

### miR-3473 was involved in TRAF3-NF-κB-TNF-α regulation axis

**Fig. 4:**
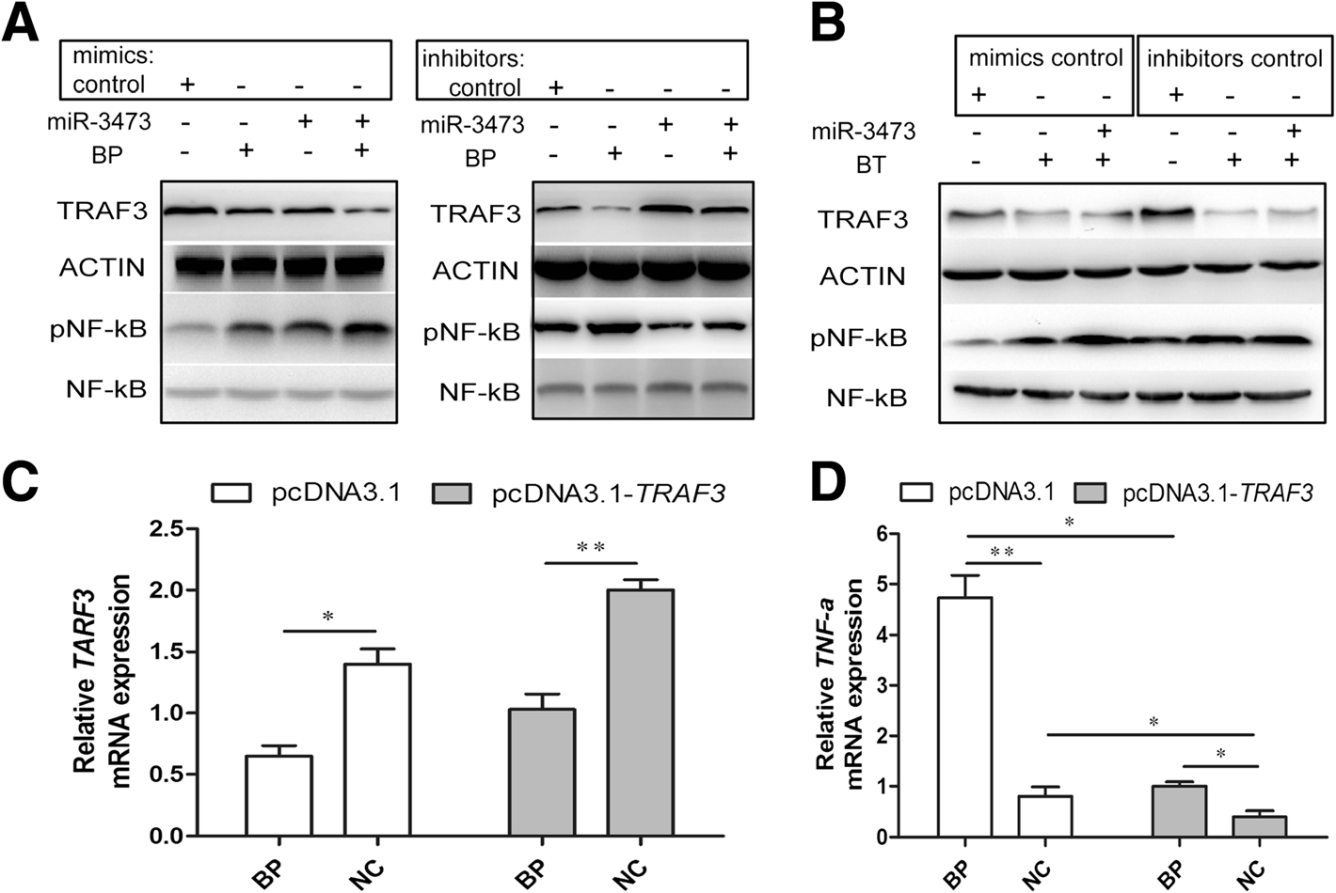
Bp infection specifically reduces TRAF3 by miR3473 which activates NF-κB-TNF-α pathway. **a** Protein levels of TRAF3, Phospho-NF-κB p65 and NF-κB p65 in Bp-infected or uninfected macrophages transfected with miR-3473 mimic, inhibitor or controls, as assessed by western blot with Actin as an internal control. **b** Protein levels in Bt-infected or uninfected RAW264.7 cell were tested as Fig. 4a proceed. **c** qRT-PCR analysis of *TRAF3* mRNA in *TRAF3*-overexpressed and negative control macrophages, with *actin* as an internal control. **d** TNF-α mRNA was evaluated in *TRAF3*-overexpressed or untransfected macrophages at 12 h after Bp infection. *P value < 0.05, **P value < 0.01

Since TRAF3 has been known as a negative regulator of NF-κB pathway, the mechanism for how miR-3473 regulates TNF-α expression is unknown. miR-3473 mimic and inhibitor were used to change the expression level of miR-3473. TRAF3 expression and NF-κB activity of macrophages treated by Bp, Bt or PBS control were evaluated by western blot (Fig. 4a&b). After transfection of miR-3473 mimics, NF-κB pathway was enhanced significantly (showed by phospho-NF-κB p65 level) with or without Bp infection (Fig. 4a). On the contrary, miR-3473 inhibitor would decrease the activity of NF-κB in Bp-infected macrophages, but not in Bt-infected macrophages (Fig. 4a&b). It suggested that there should be a miR-3473-TRAF3-NF-κB regulation axis which would play a vital role in Bp-mediated inflammatory response in macrophages.

To further clarify the regulation of TRAF3 on TNF-α expression, murine *TRAF3* coding sequence was cloned into *pcDNA3.1* plasmid and the construction was transfected into the murine macrophages, then verified by qRT-PCR (Fig. 4c). Significantly, TNF-α mRNA decreased after enhancement of *TRAF3*-UTR in macrophages (Fig. 4d). These results suggested that miR-3473 manipulated TRAF3-NF-κB-TNF-α regulation axis to affect TNF-α expression in Bp-infected macrophages.

### miR-3473 was responsible for the differences of TNF-α release and cell apoptosis between Bp and Bt

**Fig. 5:**
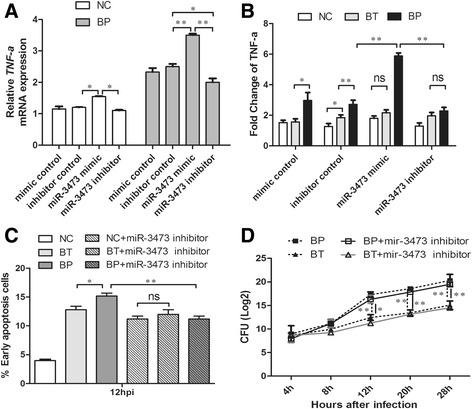
miR-3473 regulated TNF-α release specifically, but did not influence bacterial replication in Bp-treated macrophages. **a** qRT-PCR analysis of TNF-α mRNA expression in Bp-infected or uninfected macrophages pre-treated with miR-3473 mimic, inhibitor or controls. **b** TNF-α mRNA levels were measured in Bp, Bt-infected or uninfected macrophages pre-treated with microRNA oligonucleotides. **c** Early apoptosis cells were gated through flow cytometry test in macrophages pre-transfected with miR-3473 inhibitor or control at 12 h after Bp or Bt infection. **d** Intracellular bacteria loads of Bp or Bt-infected macrophages transfected with miR-3473 oligonucleotides were counted at different infectious phase (4 h, 8 h, 12 h, 20 h and 28 h). *P value < 0.05, **P value < 0.01

Based on the above results, we wondered whether miR-3473 alone can influence TNF-α mRNA expression, cell apoptosis and bacterial replication in macrophages. We found that treatment with miR-3473 mimics markedly enhanced TNF-α mRNA expression in uninfected or Bp-infected macrophages (Fig. 5a). Conversely, miR-3473 inhibitor oligonucleotides would decrease TNF-α mRNA in Bp-infected macrophages and abrogate the difference of TNF-α mRNA levels between Bp and Bt-infected cells (Fig. 5a). As Fig. 1d and Fig. 5b shown, Bt can upregulate TNF-α mRNA expression at the late infectious phase (>16 hpi), but it would not change TNF-α expression after treatment of miR-3473 mimics or inhibitors like that in Bp-treated macrophages (Fig. 5b). It was the same case for the regulation of miR-3473 on cell apoptosis in Bp or Bt-infected macrophages (Fig. 5c). This indicated that miR-3473, as an independent regulation factor, can play a key role in Bp-induced TNF-α expression and cell apoptosis.

Given that it was different for intracellular growth rate between Bp and Bt (shown in Fig. 1a), we tested whether miR-3473 affected bacterial survival in macrophages. Intracellular bacteria load was determined through culture on LB plate at different time points (from 4 hpi to 28 hpi). However, miR-3473 inhibitors had no obvious effect on the intracellular growth of Bp or Bt (Fig. 5d). It suggested that miR-3473 can manipulate bacteria-induced host inflammatory but do not influence the recycle of intracellular Bp or Bt.

### miR-3473 was induced significantly in lung cells from Bp-infected mice but would not affect murine survival

**Fig. 6:**
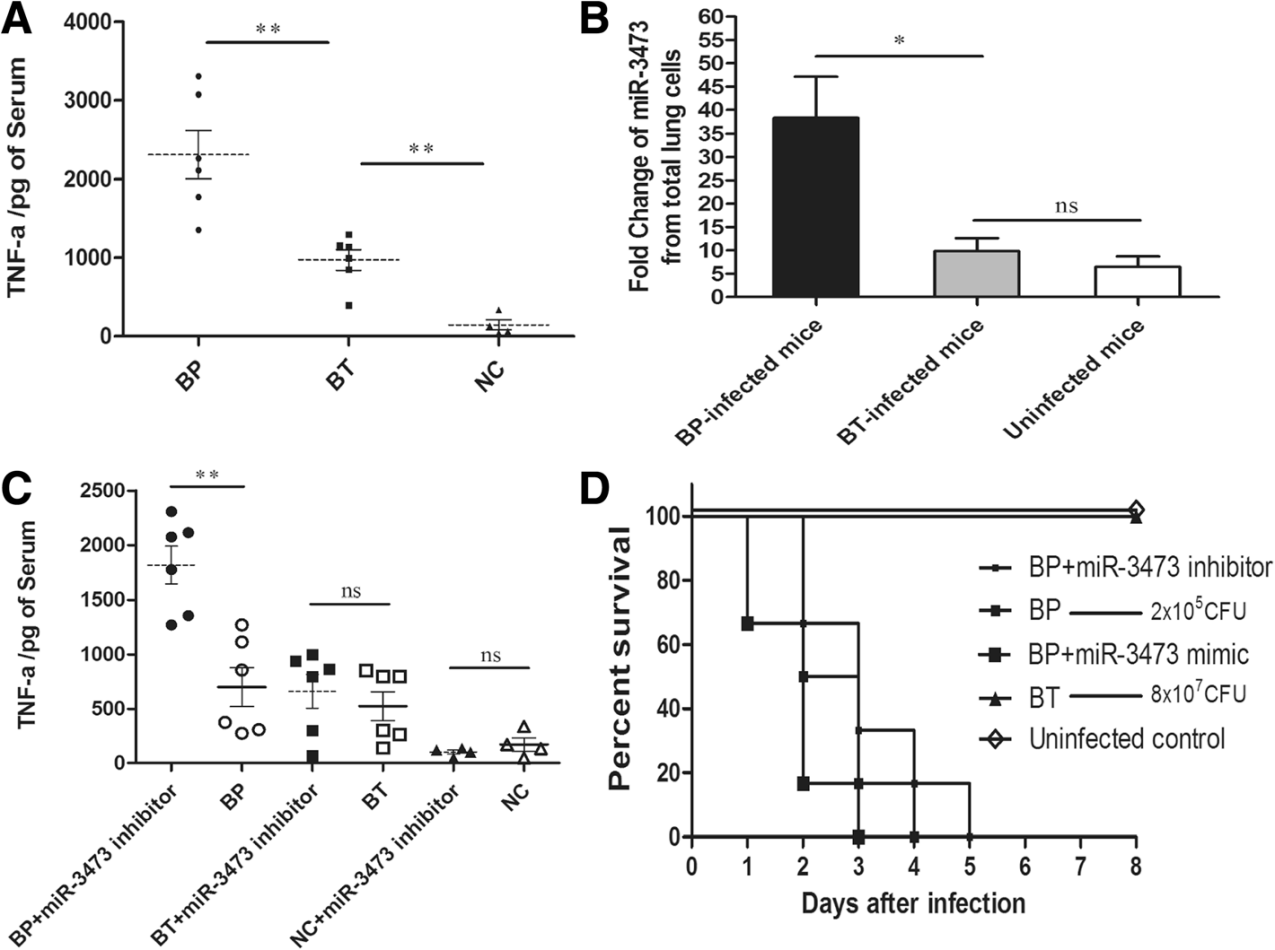
miR-3473 expressed significantly in lung cells from Bp-infected mice, involved in the TNF-α release and animal survival. **a** TNF-α release was measured by ELISA on blood samples from Bp, Bt-infected or uninfected mice on the fourth day after infection (4 dpi, with the dose of 2 × 10^5^ CFU Bp or 8 × 10^7^ CFU Bt or no bacteria, both in 50 μL PBS and injected into mice). **b** Lung cells were separated from Bp-infected, Bt-infected or uninfected mice at 4 dpi. and miR-3473 expression was measured by qRT-PCR described as above. **c** miR-3473 inhibitor oligonucleotide was administrated to mice (i.n., through breathing) on the day before inoculation and TNF-α release was measured at 4 dpi. **d** Survival curves of mice infected with Bp, Bt or PBS for 7 days with or without treatment of miR-3473 inhibitor oligonucleotides. *P value < 0.05, **P value < 0.01

In vivo test showed that TNF-α level was higher in blood of Bp-infected mice compared to that of Bt-infected or uninfected mice (Fig. 6a). Previous studies and above results both suggested that TNF-α was an important inflammatory factor associates with difference between Bp and Bt infectious process. As described in material and method, qRT-PCR was applied to test the expression of miR-3473 in murine lung cells. We found that miR-3473 was induced higher in lung cells from Bp-infected mice than that from Bt-infected or uninfected mice (Fig. 6b). In addition, miR-3473 inhibitor was administered into mice and its regulation on TNF-α release was equally obvious like that in vitro (Fig. 6c). Furthermore, miR-3473 had a detrimental impact on the survival period of mice infected with lethal dose of Bp (Fig. 6d). These results suggested miR-3473 was indulgent for excessive inflammatory response and associated with acute death of mice after lethal Bp infection.

## Discussion

Melioidosis is notorious for its complex nature, resistance to antibiotics and poor outcomes and has been an unexpected threat to public health and caused heavy economic burden in endemic regions. The pathogen, *Burkholderia pseudomallei* (Bp), has been researched for many years but the effective way against intracellular replication, resistance and serious clinical outcomes is still unavailable since several possible vaccines are underway [25]. It is widely distributed and endemic in tropical regions, including southeast Asia and southern Australia [26]. The increasing number of sporadic cases have been reported around the world, showing the dissemination of this pathogen beyond the original scope [27]. Its homologous and avirulent species, *Burkholderia thailandensis* (Bt), a Bp-like and avirulent specie, has been also classified as an opportunistic bacteria and found related with human pulmonary cystic fibrosis [11]. Bt has been investigated for possible vaccine candidate against melioidosis or taken as a model for melioidosis research [6].

Some previous studies have compared Bp and Bt, their intracellular phenotypes and associated host inflammatory response [4, 7, 8, 10, 13]. Wand et al. [28] observed an inverse association with their macrophage cytotoxicity and the virulence in mice after Bt infected. While in this study, we found that Bp’s cytotoxity in cell (at late infectious phases) and lethality to mice were both stronger than Bt’s. This difference may associate with different using of bacterial strain, infectious dose or testing time point. Also, what we focus is about the different inflammatory response in murine macrophages treated by Bp and Bt in vitro and the reasons behind. Since the inflammatory response is more complicated than that in cell infectious models. Based on comparative genomic analysis, metabolic and virulence differences between these two species have been ascribed to their divergent gene clusters, including capsular polysaccharides and the Type III secretion needle complex. Recently, it has been reported that inhalation of Bt also could result in lethal necrotizing pneumonia and death in mice with a dose of 10^5^ CFU/lung [6]. Considering these findings, Bt could not be simply taken as an innocuous agent, but a Bp-like opportunistic pathogen, with remarkable intracellular expansion and cytotoxicity. Therefore, it is meaningful to study the mechanism between Bp and Bt and their related cellular process, which may help us to understand their difference of virulence and provide promotion for vaccine development with avirulent strain from suspected pathogen.

Recently years, microRNA screening has been applied to explore the mechanism for bacterial infectious process and related host defence [29, 30]. This study also identified a specific microRNA associating with differences of Bp and Bt infectious process, especially on the inflammatory response of Bp and Bt-infected cells or animals. With a same infective dose, Bp would induce higher TNF-α release and apoptosis levels compared to Bt, especially at the late infection phase. Based on microRNA chip analysis, several highly expressed microRNAs were found from Bp-infected cells compared to Bt-infected cells. Among them, miR-3473 was found to be specific to Bp infection and significantly induced along with the infection process both in murine macrophages and pulmonary cell line (TC-1). In addition, we found that miR-3473 was able to affect the TNF-α release, cell apoptosis and inflammatory response via a miR-3473-*TRAF3*-TNF-α network. However, there was no direct effect on intracellular bacterial replication. It suggested that these oligonucleotides could manipulate biological procedure and influence intercellular bacterial growth or host response indirectly.

In vivo, it was also a similar case. As described above, miR-3473 expressed significantly and specifically in total lung cells of mice infected with Bp. On the contrary, after intravenous administration with miR-3473 inhibitor, the survival period of Bp-infected mice extended although the death rate could not be altered at the end. Temperately, we attributed this protection effect to the inhibition of TNF-α release. Actually, it is probable that there are some other influence factors involved in these biochemical events.

Additionally, we have found some other different phenotypes between Bp and Bt-treated macrophages. For example, Bp induced more autophagosomes (named as LC3-associated phagocytosis because of the unique single autophagosome structure) compared to Bt. Recently, we have proved that Bp-associated regulation on host autophagy relates to some bacteria-induced microRNAs [19]. miRNA chips screening may uncover more hidden network associating with some special miRNAs or specific virulence factors and find the reason for those differences of virulence, intracellular persistence and infection outcomes between virulent and avirulent pathogens [31].

## Conclusion

*Burkholderia pseudomallei* (Bp) and *Burkholderia thailandensis* (Bt) are both belonging to *Burkholderia spp.* As a Bp-like specie, Bt has many similar phenotypes to Bp, including replication capacity in various type of cells, cytotoxicity to host cells and inflammatory responses, maybe only differing in the degree or intensity. It was found that Bt replicated slower in macrophages, inducing slighter apoptosis and inflammatory reaction compared to Bp. In vivo, Bp is highly lethal but Bt is totally not lethal even at a very high dose. As our gene chips analysis shown, TNF-α expression showed an extremely high degree in Bp-infected murine macrophages, which have been proved to associate with a bad outcome of melioidosis patients in clinic. From a view of upper stream of regulation, rather than bacterial T3SS-associated factor previously reported, we explored microRNA-mRNA network to find the role of miRNAs for bacteria-host interaction, including different inflammatory responses between pathogens. Through microRNA screening, we found a significantly changed microRNA (mmu-miR-3473) raised along with TNF-α release and cell apoptosis after Bp infection (compared to Bt). In vivo, the miR-3473-*TRAF3*-TNF-α network was also regulating the TNF-α release and the survival of mice. This is a first research on the mechanism for host responses after Bp or Bt infection from a view of microRNA. In the future, more microRNA-associated regulation networks would be revealed and shed more light on the pathogenesis research on differences between virulent and avirulent pathogens.

## Abbreviations

Bp: *Burkholderia pseudomallei, B. pseudomallei*
Bt: *Burkholderia thailandensis, B. thailandensis*
cfu/CFU: Colony-forming unit
dpi: Days post infection/inoculation
hpi: h.p.i., hours post infection
MNGC: Multinucleated giant cell
MOI: Multiplicity of infection
NF-κB: Nuclear factor kappa-light-chain-enhancer of activated B cells
TRAF3: TNF receptor-associated factor 3
UTR: Untranslated region

## References

[1] Hantrakun V, Chierakul W, Chetchotisakd P, Anunnatsiri S, Currie BJ, Peacock SJ, Day NP, Cheah PY, Limmathurotsakul D, Lubell Y (2015). Cost-effectiveness analysis of parenteral antimicrobials for acute melioidosis in Thailand. Trans R Soc Trop Med Hyg.

[2] Brett PJ, Deshazer D, Woods DE (1997). Characterization of Burkholderia pseudomallei and Burkholderia pseudomallei-like strains. Epidemiol Infect.

[3] Brett PJ, DeShazer D, Woods DE (1998). Burkholderia thailandensis sp. nov., a Burkholderia pseudomallei-like species. Int J Syst Bacteriol.

[4] Yu Y, Kim HS, Chua HH, Lin CH, Sim SH, Lin D, Derr A, Engels R, DeShazer D, Birren B (2006). Genomic patterns of pathogen evolution revealed by comparison of Burkholderia pseudomallei, the causative agent of melioidosis, to avirulent Burkholderia thailandensis. BMC Microbiol.

[5] Ngugi SA, Ventura VV, Qazi O, Harding SV, Kitto GB, Estes DM, Dell A, Titball RW, Atkins TP, Brown KA (2010). Lipopolysaccharide from Burkholderia thailandensis E264 provides protection in a murine model of melioidosis. Vaccine.

[6] Haraga A, West TE, Brittnacher MJ, Skerrett SJ, Miller SI (2008). Burkholderia thailandensis as a model system for the study of the virulence-associated type III secretion system of Burkholderia pseudomallei. Infect Immun.

[7] Ngamdee W, Tandhavanant S, Wikraiphat C, Reamtong O, Wuthiekanun V, Salje J, Low DA, Peacock SJ, Chantratita N. Competition between Burkholderia pseudomallei and B. thailandensis. BMC Microbiol. 2015;15:56. 10.1186/s12866-015-0395-7 PMC4365494 25879538

[8] Bartell JA, Yen P, Varga JJ, Goldberg JB, Papin JA (2014). Comparative metabolic systems analysis of pathogenic Burkholderia. J Bacteriol.

[9] Kespichayawattana W, Rattanachetkul S, Wanun T, Utaisincharoen P, Sirisinha S (2000). Burkholderia pseudomallei induces cell fusion and actin-associated membrane protrusion: a possible mechanism for cell-to-cell spreading. Infect Immun.

[10] Charoensap J, Utaisincharoen P, Engering A, Sirisinha S (2009). Differential intracellular fate of Burkholderia pseudomallei 844 and Burkholderia thailandensis UE5 in human monocyte-derived dendritic cells and macrophages. BMC Immunol.

[11] Lertpatanasuwan N, Sermsri K, Petkaseam A, Trakulsomboon S, Thamlikitkul V, Suputtamongkol Y (1999). Arabinose-positive Burkholderia pseudomallei infection in humans: case report. Clin Infect Dis.

[12] Glass MB, Gee JE, Steigerwalt AG, Cavuoti D, Barton T, Hardy RD, Godoy D, Spratt BG, Clark TA, Wilkins PP (2006). Pneumonia and septicemia caused by Burkholderia thailandensis in the United States. J Clin Microbiol.

[13] Wongprompitak P, Sirisinha S, Chaiyaroj SC (2009). Differential gene expression profiles of lung epithelial cells exposed to Burkholderia pseudomallei and Burkholderia thailandensis during the initial phase of infection. Asian Pac J Allergy Immunol..

[14] West TE, Chantratita N, Chierakul W, Limmathurotsakul D, Wuthiekanun V, Myers ND, Emond MJ, Wurfel MM, Hawn TR, Peacock SJ (2013). Impaired TLR5 functionality is associated with survival in melioidosis. J Immunol.

[15] Suputtamongkol Y, Kwiatkowski D, Dance DA, Chaowagul W, White NJ (1992). Tumor necrosis factor in septicemic melioidosis. J Infect Dis.

[16] Tufekci KU, Oner MG, Meuwissen RL, Genc S (2014). The role of microRNAs in human diseases. Methods Mol Biol.

[17] Ullah S, John P, Bhatti A (2014). MicroRNAs with a role in gene regulation and in human diseases. Mol Biol Rep.

[18] Ordas A, Hegedus Z, Henkel CV, Stockhammer OW, Butler D, Jansen HJ, Racz P, Mink M, Spaink HP, Meijer AH (2011). Deep sequencing of the innate immune transcriptomic response of zebrafish embryos to Salmonella infection. Fish Shellfish Immunol.

[19] Li Q, Fang Y, Zhu P, Ren CY, Chen H, Gu J, Jia YP, Wang K, Tong WD, Zhang WJ, et al. Burkholderia pseudomallei survival in lung epithelial cells benefits from miRNA-mediated suppression of ATG10. Autophagy. 2015;11(8):1293–1307. 10.1080/15548627.2015.1058474 PMC4590646 26151773

[20] Westermann AJ, Gorski SA, Vogel J (2012). Dual RNA-seq of pathogen and host. Nat Rev Microbiol.

[21] Fang Y, Huang Y, Li Q, Chen H, Yao Z, Pan J, Gu J, Tang B, Wang HG, Yu B (2012). First genome sequence of a Burkholderia pseudomallei Isolate in China, strain BPC006, obtained from a melioidosis patient in Hainan. J Bacteriol.

[22] Rosas-Taraco AG, Higgins DM, Sanchez-Campillo J, Lee EJ, Orme IM, Gonzalez-Juarrero M (2009). Intrapulmonary delivery of XCL1-targeting small interfering RNA in mice chronically infected with Mycobacterium tuberculosis. Am J Respir Cell Mol Biol.

[23] Guan K, Wei C, Zheng Z, Song T, Wu F, Zhang Y, Cao Y, Ma S, Chen W, Xu Q (2015). MAVS Promotes Inflammasome Activation by Targeting ASC for K63-Linked Ubiquitination via the E3 Ligase TRAF3. J Immunol.

[24] Song YJ, Kang MS (2010). Roles of TRAF2 and TRAF3 in Epstein-Barr virus latent membrane protein 1-induced alternative NF-kappaB activation. Virus Genes.

[25] Patel N, Conejero L, De Reynal M, Easton A, Bancroft GJ, Titball RW (2011). Development of vaccines against burkholderia pseudomallei. Front Microbiol.

[26] Fang Y, Chen H, Li YL, Li Q, Ye ZJ, Mao XH (2015). Melioidosis in Hainan, China: a restrospective study. Trans R Soc Trop Med Hyg.

[27] Wiersinga WJ, van der Poll T (2009). Burkholderia pseudomallei tropism and the melioidosis road map. J Infect Dis.

[28] Wand ME, Muller CM, Titball RW, Michell SL (2011). Macrophage and Galleria mellonella infection models reflect the virulence of naturally occurring isolates of B. pseudomallei, B. thailandensis and B. oklahomensis. BMC Microbiol.

[29] Wang S, Zhang Z (2011). Maggot microRNA: A new inhibitory pathway to bacterial infection. Med Hypotheses.

[30] Ma F, Xu S, Liu X, Zhang Q, Xu X, Liu M, Hua M, Li N, Yao H, Cao X (2011). The microRNA miR-29 controls innate and adaptive immune responses to intracellular bacterial infection by targeting interferon-gamma. Nat Immunol.

[31] Benanti EL, Nguyen CM, Welch MD (2015). Virulent burkholderia species mimic host actin polymerases to drive actin-based motility. Cell.

[32] Yi Z, Stunz LL, Bishop GA (2013). TNF receptor associated factor 3 plays a key role in development and function of invariant natural killer T cells. J Exp Med.

